# Characterization of Modified Natural Minerals and Rocks for Possible Adsorption and Catalytic Use

**DOI:** 10.3390/molecules25214989

**Published:** 2020-10-28

**Authors:** Kateřina Strejcová, Zdeněk Tišler, Eliška Svobodová, Romana Velvarská

**Affiliations:** Unipetrol Centre for Research and Education, a.s, Areál Chempark 2838, Záluží 1, 436 70 Litvinov, Czech Republic; zdenek.tisler@unicre.cz (Z.T.); eliska.svobodova@unicre.cz (E.S.); romana.velvarska@unicre.cz (R.V.)

**Keywords:** natural minerals and rocks, natural zeolite, clinoptilolite, marlstone, phonolite, metakaolin, acid leaching

## Abstract

This study focused on natural materials such as clinoptilolite (CLI), metakaolin (MK), marlstone (MRL) and phonolite (PH). Clinoptilolite is one of the most known and common natural minerals (zeolites) with a unique porous structure, metakaolin is calcined kaolin clay, marlstone is a sedimentary rock and phonolite is an igneous rock composed of alkali feldspar and other minerals. These natural materials are mainly used in the building industry (additions for concrete mixtures, production of paving, gravels) or for water purification, but the modification of their chemical, textural and mechanical properties makes these materials potentially usable in other industries, especially in the chemical industry. The modification of these natural materials and rocks was carried out by leaching using 0.1 M HCl (D1 samples) and then using 3 M HCl (D2 samples). This treatment could be an effective tool to modify the structure and composition of these materials. Properties of modified materials were determined by N_2_ physisorption, Hg porosimetry, temperature programmed desorption of ammonia (NH_3_-TPD), X-ray fluorescence (XRF), X-ray powder diffraction (XRD), diffuse reflectance infrared Fourier transform (DRIFT) and CO_2_ adsorption using thermogravimetric analysis (TGA). The results of N_2_ physisorption measurements showed that that the largest increase of specific surface area was for clinoptilolite leached using 3M HCl. There was also a significant increase of the micropore volume in the D2 samples. The only exception was marlstone, where the volume of micropores was zero even in the leached sample. Clinoptilolite had the highest acidity and sorption capacity of CO_2_. TGA showed that the amount of CO_2_ adsorbed was not significantly related to the increase in specific surface area and the opening of micropores. Hg porosimetry showed that acid leaching using 0.1 M HCl and 3 M HCl resulted in a significant increase in the macropore volume in phonolite, and during leaching using 3M HCl there was an increase of the mesopore volume. From the better properties, cost-efficient and environmental points of view, the use of these materials could be an interesting solution for catalytic and sorption applications.

## 1. Introduction

Natural zeolite is a volcanic, or volcano-sediment, material composed of pores and corner-sharing aluminosilicate tetrahedrons (AlO_4_, SiO_4_), which are interconnected into three-dimensional structures. These aluminosilicate minerals with rigid anionic frameworks contain well-defined channels and cavities of different sizes. These cavities are occupied with positively charged alkali (Na^+^, K^+^), and alkali earth (Ca^2+^, Mg^2+^) ions, OH-groups or H_2_O molecules. Channels allow the movement of molecules and ions from and into the structure of the zeolite [1,2,3]. The most common natural zeolite is clinoptilolite with a high Si/Al ratio, electively exchanged with large cations such as Cs^+^, Rb^+^, K^+^, NH_4_^+^, Na^+^, Ba^2+^, and Sr^2+^ [4] (possibly also Pb^2+^, Cu^2+^, Fe^3+^, Cr^3+^, Zn^2+^) [5,6]. In our study, K,Ca-rich clinoptilolite, with a small proportion of Fe, Mg and Na ions and others in trace amounts from the East Slovak deposit, was used. The crystal chemical formula of clinoptilolite from a representative sample of the Nižný Hrabovec deposit was: |(Na_0.21_K_1.74_) (Ca_1.71_Mg_0.31_) (H_2_O)_18.28_| [Al_6.11_ Si_29.90_O_72_] [7]. Due to its adsorption capabilities, clinoptilolite is also used in a wide range of applications [8]. Zeolites can be used in water treatment, where they serve as ion-exchangers and media for slow filtration [9]. They also have application as supplementary cementitious materials [10].

Metakaolin is a poorly crystallized calcined clay formed by calcination of kaolin at temperatures of 600–800 °C and has a high pozzolanic activity that is higher than fly ash. [11,12]. Metakaolin has a higher reactivity than kaolin. Unlike the original crystalline kaolinite, the crystal lattice of metakaolin is partially decomposed by dehydroxylation. In addition to dehydroxylation, there is also delamination of the individual layers of kaolinite and the structure of [AlO_6_] units changes to [AlO_4_] [13]. Metakaolin is used as a pozzolanic additive in mortars and cements, and the geopolymer matrix for reduction of hazardous wastes and other pollutants [14].

Marlstone is a fine-grained highly porous silica sedimentary rock [15]. This rock is used not only for construction purposes but also in sculpture. As marlstone is sensitive to external influences, such as freezing/thawing, its use has been limited mainly to indoor areas [16].

Phonolite is an igneous rock, which consists mainly of alkaline feldspars and other minerals, such as analcime from the group of zeolites. It is used in the production of dark phonolite glass and tiles [17].

These natural materials are used mainly in construction, but for their wider use, e.g., for sorption and catalytic applications, it is necessary to improve their textural properties. The properties can be modified by post-treatment modifications such as dealumination, desilication or ion exchange [18]. These modifications can increase the porosity of the material and the specific surface area. Dealumination (acid leaching) removes the framework aluminum (FAl), accompanying minerals and cations and thus cleans the microporous structure. Acid leaching also increases the Si/Al ratio [19,20]. The Si/Al ratio affects properties such as ion-exchange capacity, thermal and hydrothermal stability, concentration and strength of acid sites of the Brönsted-type and activity and selectivity of catalysts. When using zeolites as catalysts, a high Si/Al ratio is favored (low aluminum content) [21].

Due to global warming and increasing greenhouse gas emissions, zeolites have also been investigated as potential CO_2_ adsorbents. However, the efficiency of adsorption is affected by their size, the form of their pores, Si/Al ratio, the number of cations and other factors [22,23]. The electric field and basicity of zeolite are related to its Si/Al ratio. The lower the Si/Al ratio, the higher the basicity, which results in higher CO_2_ adsorption [24].

The aim of this work was to compare abundant natural materials (clinoptilolite, metakaolin, marlstone and phonolite), with properties subsequently modified by acid leaching. Acid leaching, which promotes dealumination, removal of other mineral components and cations, could improve the properties of these natural materials to make them useful for catalytic and sorption applications. The effect of acid leaching of 0.1 M HCl and 3 M HCl on the above-mentioned materials was monitored and the structural and chemical properties were determined by N_2_ physisorption, Hg porosimetry, temperature-programmed desorption of ammonia (NH_3_-TPD), X-ray fluorescence (XRF), X-ray powder diffraction (XRD), diffuse reflectance infrared Fourier transform (DRIFT) and CO_2_ adsorption using thermogravimetric analysis (TGA).

These materials could be interesting alternatives for use in catalysis, especially because of their availability and low cost.

## 2. Results and Discussion

### 2.1. Characterization of Materials

**Figure 1:**
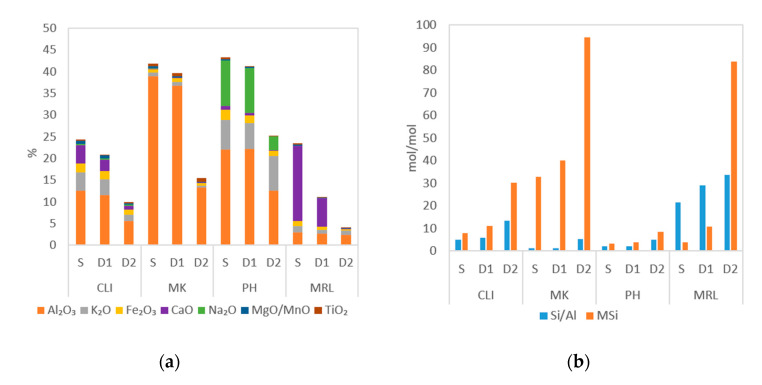
Chemical composition (silicon is up to 100%) (**a**) and Si/Al ratio and M_Si_ parameter (**b**) of clinoptilolite (CLI), metakaolin (MK), phonolite (PH) and marlstone (MRL) determined as untreated (S), leached using 0.1 M HCl (D1) and leached using 3 M HCl (D2).

**Figure 2:**
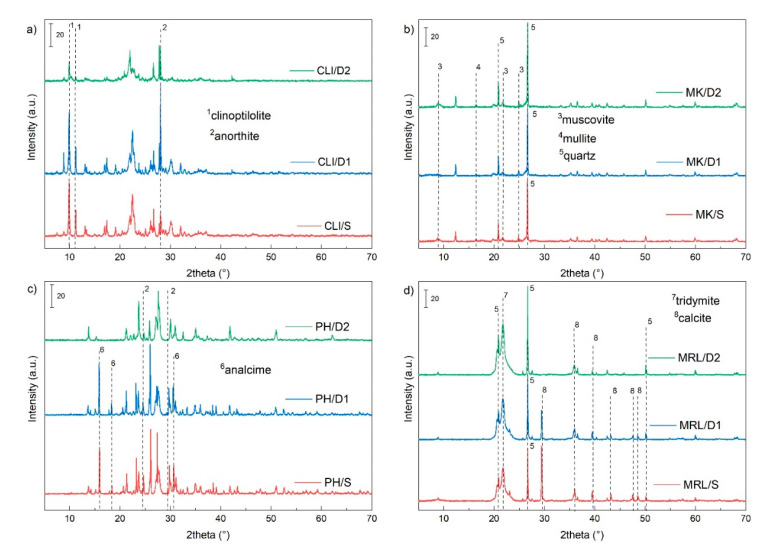
XRD pattern of clinoptilolite (**a**), metakaolin (**b**), phonolite (**c**) and marlstone (**d**).

Samples were characterized by X-ray fluorescence (XRF). Using this method, the elemental composition of the materials was determined. Figure 1a shows, that MK/S contained mainly Al and only a small amount of other components, the MRL/S sample contained mainly Ca, the sample PH/S contained a larger amount of Na and K and the CLI/S sample contained a wider spectrum of elements (i.e., K, Ca, Fe). Table S1 (Supplementary Materials) shows that leaching using 0.1 M acid (D1 samples) changed the elemental composition only slightly. The exception was the MRL/D1 sample in which there was a relatively large removal of Ca (a decrease of about 60%), present in the form of limestone (CaCO_3_) in the rock (Figure 2d). In the MRL/D2 sample, Ca was completely removed. In the CLI/D1 sample, there was a significant loss of Ca and then a partial removal of K and, as stated by Hrachovcová et al., (2020) [20] with the use of 3 M HCl (CLI/D2) there was significant removal of other elements, especially aluminum. There was also a decrease in the aluminum content and an associated increase in the Si/Al ratio in the MK/D2 and PH/D2 samples.

Figure 1b shows the Si/Al ratio and the M_Si_ parameter, which is the molar ratio of SiO_2_ to other oxides and indicates the degree of removal of components from a given material. It can be seen that with increasing strength of the acid used, the Si/Al ratio and the M_Si_ parameter also increased. The highest increase of the Si/Al ratio was in the MK/D2 sample, where the value increased four times compared to the MK/S sample. The MK/S sample contained the largest amount of Al of all the examined samples, which was removed by acid leaching. Overall, marlstone had the highest Si/Al ratio of all samples. Since this rock contains mainly CaCO_3_, and only a very small amount of Al, the Si/Al ratio increased only 1.5 times in the MRL/D2. Due to the complete removal of Ca, and the significant loss of K, Na, Mg and Fe, the M_Si_ parameter increased 22 times in the MRL/D2 sample. The smallest increase in the M_Si_ parameter occurred in phonolite. In the PH/D2 sample, acid leaching did not remove K as in the other samples. This suggests that K is tightly trapped in the mineral grid of minerals contained in phonolite.

The crystal structure was characterized by X-ray powder diffraction (XRD). Figure 2a shows a typical XRD pattern for clinoptilolite (PDF 83–1261) with intensities at 2θ 9.9°, 11.2°, 17.5° and 22.4° [25]. The XRD pattern also contained peaks belonging to cristobalite and feldspars present in relatively small amounts, as well as accessory biotite and quartz. [26]. Typical clinoptilolite peaks (2θ 9.9°, 11.2°, 17.5° and 22.4°) were most affected by dealumination using 3 M HCl, where the intensities decreased. The decrease is related to the disruption of the structure due to the removal of Al from the zeolite lattice. Using 0.1 HCl, the main peaks remained almost unchanged.

Figure 2b shows the XRD pattern of metakaolin. Metakaolin is a highly amorphous to polycrystalline material [27]. The most significant peak at 2θ 26.6° belongs to quartz (PDF 79–1910). Other less intense peaks belong to mullite (PDF 79–1458) and muscovite (PDF 74–1392) [28]. Leaching using 0.1 M HCl and 3 M HCl had almost no effect on the intensity of the peaks, nor on the most pronounced peak quartz, which remained structurally stable.

The XRD pattern of phonolite showed peaks belonging to feldspar group minerals (albite (PDF 83–1658), sanidine (PDF 75–0927), nepheline (PDF 70–1582), and anorthite (PDF 70–0287)), and to the zeolite group (analcime (PDF 72–0445)) (Figure 2c). Leaching decreased the intensities of all peaks and peaks belonging to analcime and anorthite were no more visible in the spectra of the PH/D2 sample.

The XRD pattern of marlstone is shown in Figure 2d. The two most intense peaks at 2θ 21.8°, 26.7° and 29.4° correspond to tridymite (PD F76–0894), quartz (PDF 78–2315) and calcite (PDF 05–0586), respectively. The marlstone also generally contains clay minerals (kaolinite, glauconite, illite, and/or mixed illite/smectite structures). Quartz, which is present in the marlstone, comes from original siliceous bioclasts (siliceous ooze), the remnants of which can be found in some areas [16]. Acid leaching using 0.1 M HCl decreased the intensity of the calcite peak. Furthermore, we observed that leaching using 3 M HCl completely eliminated the peak belonging to calcite and, conversely, increased the intensity of the peak belonging to quartz.

**Figure 3:**
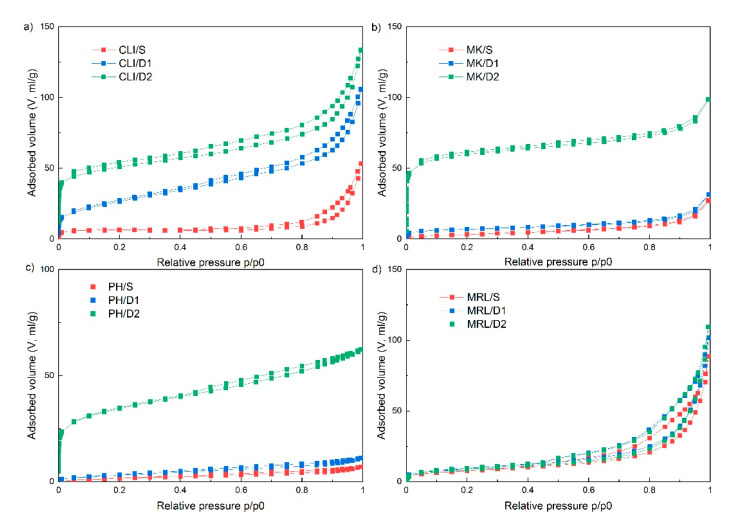
Nitrogen adsorption and desorption isotherms of clinoptilolite (**a**), metakaolin (**b**), phonolite (**c**) and marlstone (**d**).

N_2_ physisorption was used to determine specific surface area (S_BET_), total pore volume and pore distribution. Figure 3 shows the sorption isotherms. The types of adsorption isotherms were determined according to the International Union of Pure and Applied Chemistry (IUPAC) classification, which show the relation between sorption and porosity [29]. The sorption isotherms of clinoptilolite are shown in Figure 3a. The isotherms corresponded to a combination of types I and IV, which are typical for microporous and mesoporous materials, respectively. The mesoporous materials were also characterized by a hysteresis loop, which, according to the IUPAC classification, corresponded to type H3. This type of hysteresis is associated with aggregates of plate-like particles that form slit-shaped pores [30]. In the CLI/D1 and CLI/D2 samples, there was a significant increase in the total pore volume to 0.163 and 0.206 cm^3^/g (Table 1), respectively. Acid leaching of clinoptilolite also led to a significant increase in the specific surface area and the volume of mesopores and micropores, due to purification of pores that were blocked by cations and mineral components [20].

The results of the physisorption measurements also showed that MK/S and MK/D1 corresponded to the type of isotherm IV, and MK/D2 to the combination of types I and IV (Figure 3b), because there was a significant increase in the volume of micropores in D2 samples (Table 1). Metakaolin showed the type of H4 hysteresis that is typical for narrow slit-like pores [30].

There was only a slight increase in the specific surface area and pore volume of the MK/D1 sample, in contrast to clinoptilolite where the difference between the CLI/S and CLI/D1 samples was more pronounced. In the MK/D2 sample, similarly to clinoptilolite, there was a significant increase in the specific surface area up to 226.5 m^2^/g (Table 1).

The adsorption isotherm of phonolite (Figure 3c) w very similar to the isotherm of metakaolin and corresponded to type IV for samples PH/S and PH D1, and a combination of types I and IV for sample PH/D2. The hysteresis type also corresponded to type H4. A significant increase in specific surface area from 7.6 m^2^/g to 120.0 m^2^/g and micropore volume from 0 cm^3^/g to 0.024 cm^3^/g occurred only in D2 samples (Table 1).

**Figure 4:**
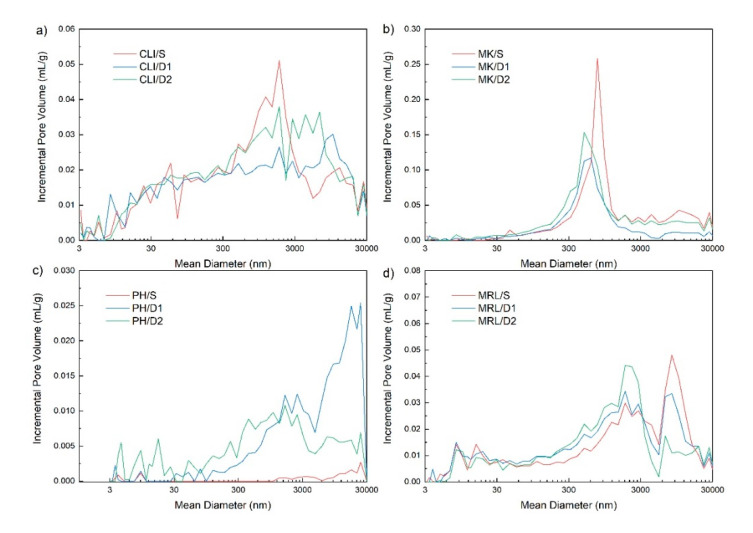
Pore distribution determined by Hg porosimetry of clinoptilolite (**a**), metakaolin (**b**), phonolite (**c**) and marlstone (**d**)**.**

Marlstone differed most from all samples (Figure 3d). MRL remained almost unchanged both after leaching using 0.1 M HCl (MRL/D1) and after leaching using 3 M HCl (MRL/D2). In the MRL/D2 sample, there was only a slight increase in the total pore volume and mesopore volume. The adsorption isotherm of marlstone corresponded to type IV, and the pronounced hysteresis loop corresponded to type H3. Compared to phonolite and metakaolin, marlstone had the largest volume of mesopores even before leaching. In contrast, as with the untreated material, the volume of micropores after leaching with 3 M HCl (MRL/D2) remained 0 cm^3^/g. This indicates the possible presence of large pores above the resolution of N_2_ physisorption (hundreds to thousands of nm), which were formed by the removal of limestone crystals from the rock structure, as shown in Figure 4d.

Macroporous structure and pore size distribution of mesopores and macropores were determined by Hg porosimetry. Clinoptilolite contained a large number of macropores up to 30 μm (Figure 4a). It can be seen from the figure that leaching using 0.1 M HCl showed a slight decrease in the volume of macropores of 300–3000 nm in the CLI/D1 sample, but a slight increase in the CLI/D2 sample. For CLI/D1 and CLI/D2 samples, there was an increase in macropores larger than 3000 nm compared to CLI/S. Metakaolin contained the largest number of macropores with a size of 300–3000 nm (Figure 4b). As with clinoptilolite, macropores were observed in the MK/D1 sample and macropores of this size were slightly increased in the MK/D2 sample. The same trend as for metakaolin was observed for marlstone (Figure 4d). Measurement of phonolite revealed that PH/S contained almost no mesopores and macropores. However, in the PH/D1 sample, there was an increase in macropores and in the PH/D2 sample there was an increase in mesopores and macropores (Figure 4c). The materials were used in the powder state, so the pores above 30,000 nm (possibly part of the pores around 3000 nm) can be attributed to the so-called false pores formed in the interparticle space.

**Figure 5:**
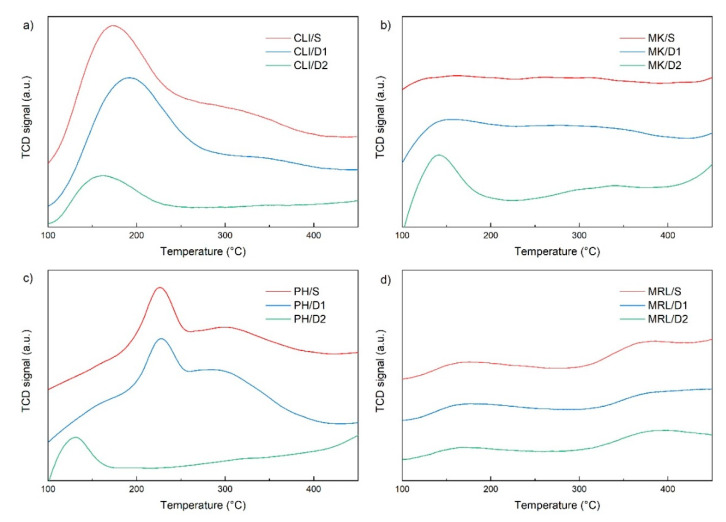
NH_3_-TPD of clinoptilolite (**a**), metakaolin (**b**), phonolite (**c**) and marlstone (**d**)**.**

For silicate materials, and their possible use in catalytic applications, the acidity and distribution of acid sites are important properties. Temperature-programmed desorption of ammonia (NH_3_-TPD) was used to determine the number of acid sites on the surface and also its strength. The low-temperature desorption peak (associated with NH_3_ desorption from weakly acidic centers–Lewis acid centers) was clearly visible in the CLI/S sample [31] with a maximum at 172 °C (Figure 5a). The temperature maximum of the CLI/D1 sample shifted slightly to higher temperatures. The CLI/D2 sample, on the other hand, shifted to lower temperatures up to 153 °C and the peak intensity decreased significantly. Furthermore, the less intense desorption peak (300 °C–350 °C) was no longer visible in the CLI/D2 sample, which was noticeable in the CLI/S and CLI/D1 samples. The CLI/D2 sample also had a higher Si/Al ratio than CLI/S and CLI/D1. The Si/Al ratio is related to Brønsted acid strength [32]. Thus, it is clear that with increasing Si/Al ratio (decreasing concentration of Brønsted acidic OH groups) the intensity of the high temperature peak decreases.

For samples MK/S and MK/D1, only slight low-temperature peaks were visible at 150 °C and 147 °C, respectively (Figure 5b). In the MK/D2 sample, there was a significant increase in concentration, and this time a significant low-temperature peak shifted to a maximum at 138 °C. There was also a slight high-temperature peak with a maximum at 312 °C.

The PH/S sample had a temperature maximum of 225 °C (Figure 5c). In the PH/D1 sample, there was only a slight shift of the temperature maximum to 229 °C, but in the PH/D2 sample, there was a significant shift of the temperature maximum up to 134 °C.

Marlstone had a low-temperature peak at 169 °C and leaching using 0.1 M HCl slightly shifted the temperature maximum to 172 °C (Figure 5d). As with clinoptilolite, the MRL/D2 sample shifted the temperature maximum back to a lower temperature at 166 °C. Figure 5d shows the second peak between 350–450 °C. When measuring a blank sample with increasing temperature and helium flow only, it was found that the second peak probably belonged to residual decomposition of the material.

In all samples, acid leaching decreased the concentration of acid sites (Table 2) and thus the total acidity. As in the study by Gao et al. (2016) [33], with increasing Si/Al ratio there was a shift of peaks to lower temperatures. The CLI/S sample had the highest value of total acidity, due to its zeolite structure. The total acidity of the CLI/S, CLI/D1 and CLI/D2 samples was several times higher than in other samples. The acidity of the modified samples decreased compared to the untreated samples. There was an increase in total acidity in MK/D1 and MK/D2 samples. This increase could be due to the entrapment of NH_3_ molecules in the micropores and thus to the insufficient elution of physisorbed molecules.

**Figure 6:**
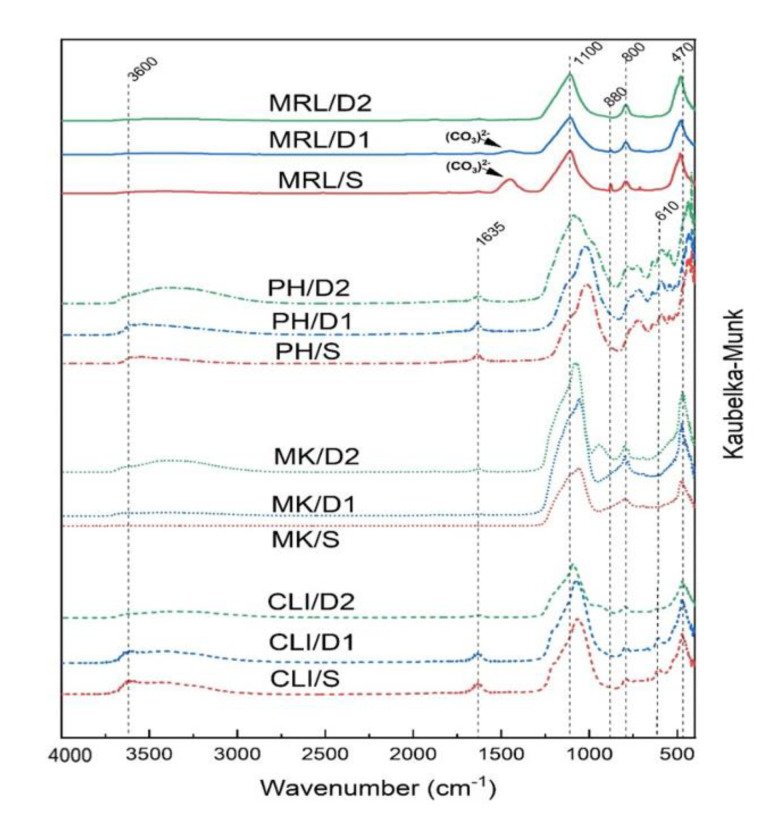
Diffuse reflectance infrared Fourier transform (DRIFT) spectra of CLI, MK, PH and MRL.

Diffuse reflectance infrared Fourier transform (DRIFT) spectra showing the structure of the samples are shown in Figure 6. Since these materials are mainly silicates, there is a significant band at 850–1300 cm^−1^ belonging to Si–O–Si antisymmetric stretching vibrations and a 470 cm^−1^ band corresponding to the Si–O–Si bending vibrations [34]. Acid leaching slightly shifted the band at about 1070 cm^−1^ to higher wave numbers. This shift is caused by dealumination and a change in the Si–O–Si angle and bond strength [35]. For clinoptilolite and phonolite, there is a noticeable shoulder around 1200 cm^−1^, which can be associated with extra framework aluminum, whose intensity decreased with the strength of the acid used [31]. A band at 800 cm^−1^, corresponding to the bend vibrations of Al–O–Si, was observed for all materials. This band remained almost unchanged during acid leaching. For clinoptilolite, a band belonging to Al–O antisymmetric stretching vibrations of AlO_4_ groups at 610 cm^−1^ was visible [36]. For the CLI/D2 sample, this band completely disappeared. DRIFT spectra showed a broad band at 3400–3700 cm^−1^ due to stretching vibration of hydroxyl (Si–OH) groups. During acid leaching, the -OH groups increased and thus the intensity of this band increased in D2 samples. A different intensity band at about 1635 cm^−1^ was also visible in the spectra, attributed to the bond vibration of water absorbed at the surface or entrapped in cavities [37]. In the DRIFT spectra of MRL/S, bands were visible at about 880 and 1420–1460 cm^−1^, typical of carbonate species [38]. In the MRL/D1 sample, there was a decrease in the intensity of these carbonate bands, and in the MRL/D2 sample the bands completely disappeared from the spectrum.

### 2.2. Adsorption Tests

Due to the rise of CO2 level in the atmosphere, one of the possibilities of using natural materials is the sorption of CO_2_. The sorption properties of the materials were investigated by CO_2_ sorption using a thermogravimetric analysis instrument (TGA). Our results, as well as the studies of Tišler et al. (2019) [18] and Yu et al. (2012) [22], show that clinoptilolite had the best sorption properties of all investigated natural materials (Table 3). The amount of CO_2_ adsorbed for the CLI/S sample was 1.8 wt%. The sorption capacity of the CLI/D1 sample increased slightly to 2.1 wt%. However, by leaching using 3 M HCl, the amount of adsorbed CO_2_ decreased to a value of 1.1 wt%. This result confirms that basicity increases with the content of Al^3+^ ions (higher content of exchangeable ions) [23]. It follows that CLI/D2, which had the highest Si/Al ratio (the lowest Al^3+^ content), had the worse adsorption capacity and selectivity for polar molecules such as CO_2_. The decrease in the amount of CO_2_ adsorbed in the CLI/D2 sample may also be due to the lower alkali content in the structure of the material, which is responsible for the formation of basic sites [18]. Thus, the amount of CO_2_ adsorbed was not significantly related to the increase in specific surface area and the opening of micropores, as shown by the results of N_2_ physisorption (Table 1).

**Figure 7:**
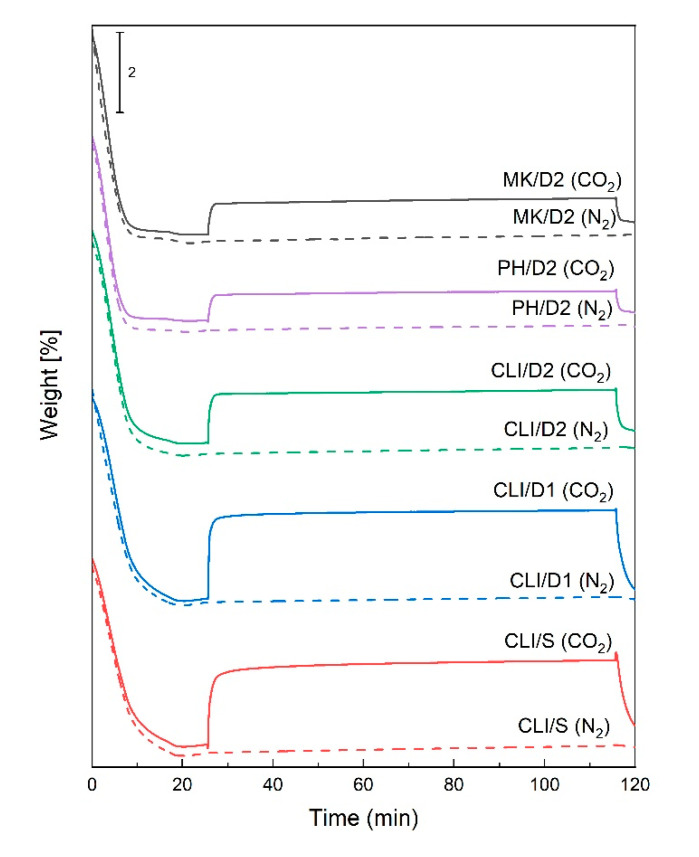
CO_2_ sorption curves of clinoptilolite determined by TGA.

It can be seen from the sorption curves in Figure 7 that for the CLI/S sample sorption occurred gradually, and for the CLI/D1 and CLI/D2 samples sorption was faster. That maximum CO_2_ was adsorbed in a shorter time in the CLI/D2 sample, could be due to a larger specific surface area and a larger pore volume due to acid leaching. Phonolite, marlstone and metakaolin showed only slight sorption, which was increased in the MK/D2 and PH/D2 samples. PH/D2 and MK/D2 samples showed significant increases compared to the PH/S and MK/S samples, but the amount of adsorbed CO_2_ was only about 55% and 65% compared to CLI/D2. Although the MK/D2 sample had a larger specific surface area than the CLI/D2 sample, the CO_2_ sorption was much lower. CO_2_ adsorption, in addition to the Si/Al ratio, is also related to the distribution, size and number and polarizing power of the exchangeable cations [23].

Although clinoptilolite is mainly used in sorption applications, there are many studies [39,40,41] that demonstrate the use of clinoptilolite in catalysis. Our previous research proved [17,42] that modified phonolite can be used in catalysis and, therefore, this material is further studied. This shows that even though these materials are not as effective in CO_2_ adsorption, they can offer interesting properties in other types of applications.

## 3. Materials and Methods

### 3.1. Materials

For this study were used natural minerals and rocks, such as powdered natural zeolite (CLI) ECO 50 from Nižný Hrabovec, Slovakia, which was delivered by Zeocem a.s., Bystré, Slovakia and which is characterized by a stable occurrence of K, Ca-rich clinoptilolite (at least 85 wt%), in association with low cristobalite, both a result of silica glass alteration. Original pyrogenic minerals in the tuffs did not exceed 20% including quartz, plagioclases and sporadically chloritized biotite. The phonolite (PH) from the quarry Želenice, Czech Republic was supplied in powder form by Keramost a.s., Most, Czech Republic. Powdered metakaolin (MK) was made from kaolin, which was mined at a quarry in Nové Strašecí, Czech Republic and delivered by České lupkové závody a.s., Nové Strašecí, Czech Republic. Powdered marlstone (MRL) (location Dobříčany, Czech Republic) was prepared by milling of rock in a Pulverisette 6 laboratory mill (500 rpm, tungsten-carbide mill segments).

### 3.2. Methods

First, the untreated clinoptilolite was denoted as CLI/S, metakaolin as MK/S, phonolite as PH/S and marlstone as MRL/S. These natural materials were modified by acid leaching using 0.1 M HCl (D1 leaching) or 3 M HCl (D2 leaching). Each powdered material was first dried overnight at 120 °C then a part of each dried sample was leached at 80 °C using 0.1 M HCl for 5 h with a weight ratio powder to solution 1:20. The samples were denoted as CLI/D1, MK/D1, PH/D1 and MRL/D1. The second part from each dried sample was leached using 3 M HCl for 5 h with the same weight ratio (powder to solution 1:20). The samples were denoted as CLI/D2, MK/D2, PH/D2 and MRL/D2. All leached samples were washed with demineralized water to neutral pH and dried overnight at 120 °C.

Properties of these natural and modified materials were determined by XRF, XRD, N_2_ physisorption, Hg porosimetry, NH_3_-TPD, DRIFT and TGA analysis.

The chemical composition of samples was determined by X-ray fluorescence analysis (XRF) of powder materials using an S8 Tiger (Bruker AXS GmbH, Karlsruhe, Germany) with an Rh cathode. The quant-express method, which is without a standard, was used for measurement. The results were analyzed using Spectra plus software (Version 3, Bruker AXS GmbH, Karlsruhe, Germany).

X-ray diffraction (XRD) analysis was used for crystal phase identification. XRD analysis was performed using a powder diffractometer D8 Advance ECO (Bruker AXS GmbH, Karlsruhe, Germany) with CuKα radiation (λ = 1.5406 Å). A resolution of 0.02° and a period of 0.5 was used. The XRD patterns were collected over a 2θ range of 5° to 70° and evaluated by Diffrac.EVA software (Bruker AXS GmbH, Karlsruhe, Germany) using the powder diffraction file database (PDF 4+ 2018, International Centre for Diffraction Data, Newtown Square, PA, USA).

Specific surface area and pore volume were determined by N_2_ physisorption measurements. For the complete removal of unwanted vapors and gases adsorbed on the sample surface, the samples were first degassed in an Autosorb iQ (Quantachrome Instruments, Boynton Beach, FL, USA) under vacuum at 110 °C for 16 h, then the isotherms (adsorption, desorption) were measured at the temperature of liquid nitrogen (−195.8 °C). Specific surface area was calculated from the linear plot (P/P_0_ range of 0.05–0.35) of the adsorption isotherm using the Brunauer, Emmett and Teller method (BET). For microporous materials, the BET surface was evaluated in a P/P_0_ range of 0.007–0.05. The pore size distribution was determined using nonlocal density functional theory (NLDFT).

Total intrusion volume and pore distribution in the range of 3–30,000 nm were determined using a Hg porosimeter AutoPore IV 9510 (Micromeritics Instrument Corporation, Norcross, GA, USA). The sample was degassed in a vacuum oven at 110 °C for 16 h. The sample was weighed into a powder penetrometer (3 mL) and placed in a low-pressure analysis port (0–345 kPa). After low pressure analysis, the penetrometer was inserted into a port for high pressure analysis (from atmospheric pressure to 414 MPa). The measurement resulted in an intrusion curve.

Total acidity of the samples was determined by ammonia temperature-programmed desorption (NH_3_-TPD). An Autochem 2950 HP instrument (Micromeritics Instrument Corporation, Norcross, GA, USA) was used for analysis. A quartz wool plug was first inserted into the quartz sample tube. The sample was then weighed into a sample tube and the tube was placed in the oven of an Autochem instrument. The sample was pretreated in a stream of He (25 mL/min) to 450 °C (10 °C/min) and cooled to 100 °C after 30 min. The sample was then saturated with 10% NH_3_ in He (25 mL/min) for 30 min. Physically bound molecules were eluted from the sample with He (25 mL/min) for 30 min. The desorption of chemically bound ammonia took place in a stream of He (25 mL/min) at increasing temperatures (100 °C–450 °C; 10 °C/min). The desorbed NH_3_ molecules in the outlet gas were detected using a thermal conductivity detector (TCD).

Diffuse reflectance infrared Fourier transform spectroscopy (DRIFT) was determined using Nicolet iS 10 (Thermo Scientific, Waltham, MA, USA) with 128 number of scans and resolution 2 cm^−1^.

The adsorption properties of the materials were studied using a TGA Discovery thermogravimetric analysis instrument series (TA Instruments, New Castle, DE, USA). The sample dried at 120 °C under a stream of N_2_ (20 mL/min) and, after cooling at 50 °C, CO_2_ adsorption was performed (gas flow 20 mL/min) for 90 min. Desorption was performed by reheating the sample to 120 °C in the N_2_ stream (20 mL/min). For comparison, an identical sample was always made in the nitrogen stream only (without CO_2_ sorption).

## 4. Conclusions

The aim of this work was to compare commonly occurring natural materials and evaluate their chemical and textural properties before and after acid leaching using 0.1 M HCl and 3 M HCl.

As the results showed, acid leaching of the input mineral raw materials can change their properties in a very significant way. Based on the obtained data, it can be stated that leaching using 0.1 M HCl did not have a significant effect on materials. There was not a significant change in chemical composition or crystalline structure, but incipient changes in texture were observed. For CLI/D1, MK/D1 and PH/D1 samples there was more than 100% increase in their specific surface area due to the release of components from their structures. In the case of leaching using 3 M HCl, significant changes in all monitored parameters were observed in the studied materials.

After leaching using 3 M HCl, the largest increase in specific surface area and micropore volume occurred in the MK/D2 sample. In contrast, the MRL/D2 sample had an increase in specific surface area of only 3 m^3^/g and the micropore volume remained 0 m^2^/g. Furthermore, samples PH/D1 and PH/D2 had very significant increases in the volume of macropores. Of all the materials examined, clinoptilolite had the highest acidity and was able to adsorb the largest amount of CO_2_. Although phonolite had only small sorption of CO_2_, together with clinoptilolite it is used in catalytic applications. In PH/D2 there was a noticeable disintegration of structure (disappearance of peaks belonging to anorthite and analcime), and in the CLI/D2 sample there was a decrease of intensities due to disturbance of its mineral structure.

Acid leaching improved some properties and made these materials potentially useful for sorption and catalytic applications. These materials have been characterized for their easy availability and low cost. The results showed significant differences caused by different mineralogy of individual rocks caused by different mechanisms of formation in the earth’s crust.

## Figures and Tables

**Table 1:** Textural properties of clinoptilolite, metakaolin, phonolite, marlstone determined by N2 physisorption measurement.

Sample	Specific Surface Area-S_BET_ (m^2^/g)	Surface Area (m^2^/g) *	Total Pore Volume (cm^3^/g) *	Micropore Volume (cm^3^/g) *	Mesopore Volume (cm^3^/g) *
CLI/S	24.6	28.7	0.082	0.005	0.055
CLI/D1	82.1	114.3	0.163	0.007	0.120
CLI/D2	186.3	297.7	0.206	0.051	0.123
MK/S	13.1	10.8	0.042	0.000	0.033
MK/D1	23.8	23.4	0.049	0.003	0.036
MK/D2	226.5	300.5	0.153	0.067	0.070
PH/S	7.6	4.9	0.011	0.000	0.009
PH/D1	13.1	9.2	0.017	0.000	0.015
PH/D2	120.0	163.9	0.096	0.024	0.067
MRL/S	28.2	26.2	0.137	0.000	0.096
MRL/D1	33.6	31.2	0.158	0.000	0.113
MRL/D2	31.2	30.1	0.170	0.000	0.120

* determined by NLDFT.

**Table 2:** The total acidity of natural materials determined by the NH_3_-TPD.

Sample	C_sum_ (mmol/g)	T_max1_ (°C)	T_max2_ (°C)
CLI/S	1.252	172	309
CLI/D1	1.100	188	337
CLI/D2	0.290	153	158
MK/S	0.053	150	-
MK/D1	0.141	147	-
MK/D2	0.112	138	-
PH	0.043	228	-
PH/D1	0.045	229	-
PH/D2	0.008	128	-
MRL	0.058	169	-
MRL/D1	0.056	172	-
MRL/D2	0.035	166	-

T_max_—temperature maximum (°C); C_sum_—the total concentration acid sites (mmol/g).

**Table 3:** Sorption properties determined by thermogravimetric analysis (TGA).

Sample	CO_2_ Adsorbed (wt%)
CLI/S	1.8
CLI/D1	2.1
CLI/D2	1.1
MK/S	0.4
MK/D1	0.2
MK/D2	0.7
PH/S	0.1
PH/D1	0.1
PH/D2	0.6
MRL/S	0.2
MRL/D1	0.1
MRL/D2	0.1

## Supplementary Materials

The following are available online, Table S1: Chemical composition of samples.
