# Supplementary material for: Characterization of Modified Natural Minerals and Rocks for Possible Adsorption and Catalytic Use
Source: Molecules. 2020 Oct 28;25(21):4989. doi: 10.3390/molecules25214989 (PMC7663305; doi:10.3390/molecules25214989)
Supplement: Supplementary file 1 [file molecules-25-04989-s001.pdf]

Supplementary Materials:

Article

Characterization of Modified Natural Minerals and Rocks for Possible Adsorption and Catalytic Use

Kateřina Strejcová *, Zdeněk Tišler, Eliška Svobodová, Romana Velvarská

Unipetrol Centre for Research and Education, a.s, Areál Chempark 2838, Záluží 1, 436 70 Litvínov, Czech Republic, zdenek.tisler@unicre.cz (Z.T.); eliska.svobodova@unicre.cz (E.S.); romana.velvarska@unicre.cz (R.V.)

* Correspondence: katerina.strejcova@unicre.cz

Academic Editors: Irina Savina and T. Jean Daou

Received: 2 October 2020; Accepted: 26 October 2020; Published: date

Table S1. Chemical composition of samples.

		SiO_2	Al_2O_3	K_2O	Fe_2O_3	CaO	Na_2O	MgO/MnO*	TiO_2	SUM	Si/Al	MSi
	S	75.30	12.60	4.26	1.91	4.28	0.30	0.78	0.23	99.66	5.07	7.95
CLI	D1	78.80	11.50	3.67	1.86	2.69	0.27	0.68	0.25	99.72	5.81	10.96
	D2	90.00	5.63	1.41	1.20	0.87	0.25	0.31	0.26	99.93	13.56	30.14
	S	57.90	38.90	0.86	0.70	0.28	0.05	0.41	0.59	99.68	1.26	32.80
MK	D1	60.30	36.80	0.84	0.82	0.12	0.03	0.35	0.64	99.89	1.39	39.94
	D2	84.40	13.30	0.40	0.60	0.10	-	0.21	0.90	99.90	5.38	94.67
	S	56.10	22.00	6.81	2.44	0.86	10.50	0.25	0.37	99.33	2.16	3.35
PH	D1	58.10	22.20	5.94	1.82	0.51	10.40	0.18	0.27	99.42	2.22	3.78
	D2	74.50	12.60	7.93	1.14	0.18	3.19	0.04	0.16	99.75	5.02	8.43
	S	76.30	3.01	1.44	1.11	17.30	-	0.38	0.23	99.77	21.51	3.73
MRL	D1	88.80	2.60	0.93	0.75	6.47	-	0.28	0.11	99.95	28.98	10.80
	D2	95.80	2.41	0.98	0.31	0.18	-	0.15	0.16	99.97	33.73	83.72

*MgO for CLI, MK and MRL, MnO for PH.
